# Lysine 53 Acetylation of Cytochrome *c* in Prostate Cancer: Warburg Metabolism and Evasion of Apoptosis

**DOI:** 10.3390/cells10040802

**Published:** 2021-04-03

**Authors:** Viktoriia Bazylianska, Hasini A. Kalpage, Junmei Wan, Asmita Vaishnav, Gargi Mahapatra, Alice A. Turner, Dipanwita Dutta Chowdhury, Katherine Kim, Paul T. Morse, Icksoo Lee, Joseph S. Brunzelle, Lisa Polin, Prabal Subedi, Elisabeth I. Heath, Izabela Podgorski, Katrin Marcus, Brian F.P. Edwards, Maik Hüttemann

**Affiliations:** 1 Center for Molecular Medicine and Genetics, School of Medicine, Wayne State University, Detroit, MI 48201, USA; viktoriiabazylianska@wayne.edu (V.B.); hkalpage@med.wayne.edu (H.A.K.); am4472@wayne.edu (J.W.); gmahapat@wakehealth.edu (G.M.); afturner@med.wayne.edu (A.A.T.); katiedcds@gmail.com (K.K.); morsepa@wayne.edu (P.T.M.); icksoolee@dankook.ac.kr (I.L.); 2 Department of Biochemistry, Microbiology, and Immunology, School of Medicine, Wayne State University, Detroit, MI 48201, USA; vaishnav@med.wayne.edu (A.V.); dipanwita@wayne.edu (D.D.C.); brian.edwards@wayne.edu (B.F.P.E.); 3 College of Medicine, Dankook University, Cheonan-si, Chungcheongnam-do 31116, Korea; 4 Life Sciences Collaborative Access Team, Center for Synchrotron Research, Northwestern University, Argonne, IL 60439, USA; j-brunzelle@northwestern.edu; 5 Department of Oncology, Karmanos Cancer Institute, Wayne State University, Detroit, MI 48201, USA; polinl@karmanos.org (L.P.); heathe@karmanos.org (E.I.H.); 6 Medical Proteomics/Bioanalytics-Center, Ruhr-University Bochum, 44789 Bochum, Germany; sprabal@gmail.com (P.S.); katrin.marcus@ruhr-uni-bochum.de (K.M.); 7 Department of Pharmacology, Wayne State University, Detroit, MI 48201, USA; ipodgors@med.wayne.edu

**Keywords:** cytochrome *c*, prostate cancer, acetylation, Warburg effect, cytochrome *c* oxidase, apoptosis, reactive oxygen species, cell signaling

## Abstract

Prostate cancer is the second leading cause of cancer-related death in men. Two classic cancer hallmarks are a metabolic switch from oxidative phosphorylation (OxPhos) to glycolysis, known as the Warburg effect, and resistance to cell death. Cytochrome *c* (Cyt*c*) is at the intersection of both pathways, as it is essential for electron transport in mitochondrial respiration and a trigger of intrinsic apoptosis when released from the mitochondria. However, its functional role in cancer has never been studied. Our data show that Cyt*c* is acetylated on lysine 53 in both androgen hormone-resistant and -sensitive human prostate cancer xenografts. To characterize the functional effects of K53 modification in vitro, K53 was mutated to acetylmimetic glutamine (K53Q), and to arginine (K53R) and isoleucine (K53I) as controls. Cytochrome *c* oxidase (COX) activity analyzed with purified Cyt*c* variants showed reduced oxygen consumption with acetylmimetic Cyt*c* compared to the non-acetylated Cyt*c* (WT), supporting the Warburg effect. In contrast to WT, K53Q Cyt*c* had significantly lower caspase-3 activity, suggesting that modification of Cyt*c* K53 helps cancer cells evade apoptosis. Cardiolipin peroxidase activity, which is another proapoptotic function of the protein, was lower in acetylmimetic Cyt*c*. Acetylmimetic Cyt*c* also had a higher capacity to scavenge reactive oxygen species (ROS), another pro-survival feature. We discuss our experimental results in light of structural features of K53Q Cyt*c*, which we crystallized at a resolution of 1.31 Å, together with molecular dynamics simulations. In conclusion, we propose that K53 acetylation of Cyt*c* affects two hallmarks of cancer by regulating respiration and apoptosis in prostate cancer xenografts.

## 1. Introduction

Cytochrome *c* (Cyt*c*) is a globular 104 amino acid heme protein that orchestrates cellular life and death decisions. It functions as an electron carrier in the electron transport chain (ETC) and as a trigger of apoptosis when released from the mitochondria [1,2]. Cyt*c* transfers single electrons from complex III (*bc*_1_ complex) to complex IV (cytochrome *c* oxidase, COX) where oxygen is reduced to water in the ETC. Cyt*c* is an indispensable molecule for ATP production and cell survival. Cyt*c* also acts as a signaling molecule for apoptosis upon release from the mitochondria, by interacting with apoptosis protease activating factor-1 (Apaf-1) and forming an active apoptosome, which initiates caspase-9 activity and the downstream caspase cascade. Reactive oxygen species (ROS) are a known trigger of intrinsic apoptosis. Being an electron carrier, Cyt*c* plays a role in ROS scavenging and ROS formation. In addition, Cyt*c* acts as a catalyst for cardiolipin peroxidation [1,2], which facilitates the release of Cyt*c* from the mitochondria, promoting apoptosis [3,4,5].

Cyt*c* is an ideal target of cancer cell signaling due its two primary functions in oxidative phosphorylation (OxPhos) and cell death via intrinsic apoptosis [6]. Most cancer cells rely more heavily on aerobic glycolysis instead of OxPhos, a phenomenon referred to as the “Warburg effect”. Cancer cells undergo metabolic reprogramming in order to promote anabolic biomass production needed for cell proliferation [7]. Deregulation of cellular energetics and resistance to cell death are both major hallmarks of cancer [8]. Cyt*c* is a protein that is at the intersection of pathways leading to both these mechanisms of carcinogenesis. We have recently shown that Cyt*c* is posttranslationally modified in vivo by phosphorylation in mammalian heart [9], liver [10], kidney [11,12], and brain [13] under normal conditions on five distinct residues, and that these modifications decisively regulate respiration and apoptosis. To date, no study has investigated how structural and functional changes of Cyt*c* may affect tumor metabolism in cancer in general or in prostate cancer specifically.

According to the National Cancer Institute, in 2020 there were an estimated 191,930 new cases of prostate cancer, making it the most common cancer in men. In addition, there were about 33,330 deaths due to prostate cancer, making it the second leading cause of cancer related death in men in the US [14]. Prostate cancer can be hormone responsive, where castration leads to tumor regression. Androgen deprivation therapy (ADT) is the standard of care for prostate cancer. Cancers that respond to ADT are called castrate-sensitive tumors versus castrate-resistant tumors, which resist ADT and are more aggressive and metastatic [15]. Prostate cancer is highly heterogeneous, and little is known about the role of metabolic reprogramming in disease progression [16]. Interestingly, mitochondrial dysfunction is known to be associated with prostate cancer aggressiveness [17].

In this study, we analyzed the posttranslational modifications (PTMs) of Cyt*c* in 4 castrate-resistant and 4 castrate-sensitive prostate tumor xenografts. We identified that Cyt*c* was acetylated on lysine 53 (K53) in all 8 xenografts. Cyt*c* is a highly basic protein that contains 18 lysine residues, which account for about 17% of the protein. We propose that the specific identification of a single modified residue, via K53 acetylation on Cyt*c*, is important and relevant to prostate cancer pathogenesis. Therefore, we characterized K53 acetylation in vitro using an acetylmimetic glutamine mutant Lys53Gln (K53Q) along with non-acetylated WT, Lys53Arg (K53R), and nonpolar Lys53Ile (K53I) Cyt*c*. Our experiments show that the K53Q acetylmimetic replacement of Cyt*c* promotes Warburg metabolism by lowering COX activity, evades apoptosis by lowering caspase-3 activity and cardiolipin peroxidase activity, and by increasing ROS scavenging.

## 2. Methods

### 2.1. Prostate Cancer Xenografts and Mass Spectrometry

Androgen-independent PC3 cells derived from a bone metastasis of a high-grade adenocarcinoma, and androgen-sensitive VCaP cells derived from a vertebral metastasis, were purchased from American Type Culture Collection (ATCC; Manassas, VA, USA). Cells were cultured in DMEM supplemented with 10% FBS, 10 mM HEPES, and 100 U/mL penicillin-streptomycin in a 37 °C humidified incubator ventilated with 5% CO_2._ Both cell lines were authenticated by the WSU Genomics facility and routinely tested for mycoplasma using MycoFluor Mycoplasma Detection Kit (Thermo Fisher Scientific, Waltham, MA, USA) and LookOut Mycoplasma PCR Detection Kit (Sigma-Aldrich, St. Louis, MO, USA). All experiments involving mice were performed in accordance with the protocol approved by the Institutional Animal Investigational Committee of Wayne State University and NIH guidelines. Subcutaneous injections of PC3 and VCaP cell suspensions (in PBS/Cultrex, R&D systems, Minneapolis, MN, USA) into SCID mice were performed by the Animal Model and Therapeutics Evaluation Core (AMTEC) at Wayne State University. The resulting tumors were harvested and immediately snap-frozen in liquid nitrogen and stored at −80 °C. Cyt*c* was enriched by immunoprecipitation as previously described [11].

After immunoprecipitation, enriched samples were run on a 10% tris-tricine gel. Bands corresponding to ~12 kDa were cut. Protein digestion from the gel pieces was performed by addition of trypsin (Serva Electrophoresis, Heidelberg, Germany) in a 1:50 (trypsin:protein) ratio at 37 °C for 16 h. Digestion was halted with the addition of 1:1 (*v*/*v*) solution of acetonitrile (MeCN, Sigma-Aldrich Chemie, Schnelldorf, Germany) and 0.1% trifluoroacetic acid (TFA, Sigma-Aldrich). The samples were sonicated for 10 min twice, and the supernatants were dried in a rotational vacuum centrifuge (SpeedDry RVC 2-25 Cdplus, Martin Christ Gefriertrocknungsanlagen GmbH, Osterode, Germany). The dried samples were resuspended in 0.1% TFA and the peptide amount was calculated by amino acid analysis. Briefly, the samples were dried in a glass vial and hydrolyzed (150 °C, 1 h in helium) using 6 M HCl (Sigma-Aldrich Chemie, Schnelldorf, Germany). Derivatization (56 °C, 10 min) was performed with a reagent containing 10 pmol Norvaline (Waters, Manchester, UK). The individual amino acids were quantified on an ACQUITY-UPLC system (Waters, Manchester, UK) using norvaline as an internal calibrant. The peptides were separated on an Ultimate 3000 RSLC nano system (Dionex, Idstein, Germany), containing a C18 trap column (100 μm × 2 cm, particle size 5 μm, pore size 100 Å, flow rate 30 µL/min, Thermo Fisher Scientific, Bremen, Germany), and an analytical C18 column (75 μm × 50 cm, particle size 2 μm, pore size 100 Å, flow rate 0.4 µL/min, Thermo Fisher, Bremen, Germany), which was coupled to an Orbitrap Elite mass spectrometer (Thermo Fisher Scientific). Solvent (A): 0.1% formic acid in H_2_O and solvent (B): MeCN, 0.1% formic acid in H_2_O were used for separation of the peptides, where the gradient started with 4% B (5 min) to 40% B (95–100 min) and finally returning to 4% B at 120 min. For MS measurements, capillary flow (275 °C, 1500 V) was used and full spectra between 300 and 2000 *m*/*z* (resolution 60,000 at 200 *m*/*z*) were recorded. Automated gain control of 1e6 and maximum injection time of 500 ms were set. For internal calibration, polydimethylcyclosiloxane (*m*/*z* 445.120) was used. The peptides were fragmented in a high-energy collisional dissociation cell with a normalized collision energy of 35%, a minimum mass of 130 *m*/*z* and an isolation window of ±1 *m*/*z*. An automated gain control of 1e4 and maximum injection time of 100 ms were set for the MS/MS analyses of the fragmented peptides.

For the identification of peptide-modifications, the raw MS files were analyzed using Proteome Discoverer 1.4 software (Thermo Fisher Scientific) with the Mascot V2.3 search algorithm (Matrix Science, London, UK) [18]. Searches were performed against the Uniprot/Swissprot database. Search parameters were peptide mass 400–10,000 Da, mass tolerance of 5 ppm, fragment mass tolerance of 20 amu, a maximum of 2 uncleaved sites, dynamic oxidation (methionine), dynamic methylation (lysine, C-term), and dynamic acetylation (lysine, N-term). After algorithmic identification, assignments were manually verified.

### 2.2. Immunoprecipitation and Western Blotting

Frozen PC3 and VCaP xenografts, normal human kidney and kidney cancer tissues, lung cancer tissues, and normal human prostate and prostate cancer tissues were cut into small pieces on dry-ice, lysed with homogenization buffer (50 mM Tris-Cl, pH 7.5, 1 mM EGTA, 1% Triton-X 100, 1 mM sodium orthovanadate, 50 mM KF, 5 mM sodium pyrophosphate, 0.27 M sucrose), supplemented with a protease inhibitor cocktail (#P8340, Sigma-Aldrich), and homogenized using a Teflon Dounce homogenizer by applying 60 strokes. Homogenates were centrifuged at 22,000× *g* at 4 °C for 30 min and the total protein concentration of the tissue lysates were quantitated using the DC protein assay kit (BioRad, Hercules, CA, USA). Cyt*c* was immunoprecipitated from 1 mg total protein lysate with 2 µg mouse anti-Cyt*c* antibody (#556432, clone 6H2.B4, BD Pharmingen, San Jose, CA, USA) along with anti-mouse IgG1 kappa isotype antibody as a control (# 14-4714-81, Thermo Fisher Scientific). Similarly, Cyt*c* was immunoprecipitated from cultured PC3, VCaP, and MDA-MB-231 cells with minor modification. Briefly, the cells were harvested by scraping and lysed by sonication in lysis buffer (50 mM Tris-Cl, pH 7.5, 1 mM EGTA, 1% Triton-X 100, 1 mM sodium orthovanadate, 50 mM KF, 5 mM sodium pyrophosphate). Immunoprecipitated Cyt*c* samples together with recombinant human Cyt*c* (1 µg) were resolved on a 10% tris-tricine gel and transferred onto a pre-wet PVDF membrane. The blots were probed with a 1:1,000 dilution of HRP-conjugate acetylated-lysine (Ac-K-100) MultiMab rabbit mAb mix (#6952, CST, Danvers, MA, USA) in 5% BSA or mouse anti-Cyt*c* antibody with a 1:4000 dilution (#556433, clone 7H8.2C12, BD Pharmingen) in 5% milk, followed by 1:10,000 dilution of anti-mouse IgG conjugated with HRP (#NA93IV, GE Healthcare, Chicago, IL, USA). Signal was obtained after addition of HyGLO ECL reagent (#E-2500, Denville Scientific Inc., Metuchen, NJ, USA).

### 2.3. Generation of Cytc Variants

WT rodent Cyt*c* (mouse/rat Cyt*c* protein sequences are identical), cloned into the bacterial expression plasmid pLW01 [19], was used to generate non-acetylated K53R, acetylmimetic K53Q, and nonpolar K53I Cyt*c* variants using the QuikChange II site-directed mutagenesis kit (Agilent technologies, Santa Clara, CA, USA). The following mutagenesis primers were used to amplify the plasmid. K53R forward: 5′-CTT ACA CAG ATG CCA AC**A GG**A ACA AAG GTA TCA CC-3′, K53R reverse: 5′-GGT GAT ACC TTT GTT **CCT** GTT GGC ATC TGT GTA AG-3’, K53Q forward: 5′-CTT ACA CAG ATG CCA AC**C AG**A ACA AAG GTA TCA CC-3′, K53Q reverse: 5′-GGT GAT ACC TTT GTT **CTG** GTT GGC ATC TGT GTA AG-3′, K53I forward: 5′-CTT ACA CAG ATG CCA AC**A TT**A ACA AAG GTATCA CCT G-3′, K53I reverse: 5′-CAG GTG ATA CCT TTG TT**A AT**G TTG GCA TCT GTG TAA G-3′. The PCR product was incubated for one hour at 37 °C with DpnI endonuclease (QuikChange II site-directed mutagenesis kit) to digest the methylated parental DNA, leaving the newly synthesized mutated DNA intact. The mutant plasmids were then transformed into XL10-Gold ultracompetent cells (QuikChange II site-directed mutagenesis kit) according to manufacturer’s protocol. Single bacterial colonies were picked for each mutant and all plasmids from overnight cultures were purified using QIAprep spin miniprep kit (#27106, Qiagen, Valencia, CA, USA) following the manufacturer’s protocol. NanoDrop 1000 spectrophotometer (Thermo Fisher Scientific) was used to quantify and assess the purity of DNA. The Cyt*c* mutant plasmids were confirmed by sequencing.

### 2.4. Bacterial Overexpression and Purification of Cytc Variants

Sequence-confirmed pLW01 mutant plasmids were transformed into competent C41(DE3) *E.* coli cells (#60442-1, Lucigen, Middleton, WI, USA) for protein overexpression and purification, using the heat shock method as described in the manufacturer’s protocol. Transformed bacteria were plated on LB-agar plates containing 100 µg/mL ampicillin. Selected clones were inoculated in 10 mL of LB medium with 100 µg/mL ampicillin and cultured overnight at 37 °C. The overnight cultures were transferred into 1L of terrific broth medium (Research Products International, Mount Prospect, IL, USA) with 100 µg/mL of carbenicillin. The cultures were grown until an OD_600_ of 1 was reached, and 100 µM IPTG was added to induce Cyt*c* expression. The induced cultures were incubated for 6 h to allow protein expression and the bacterial cells were pelleted by centrifugation at 8400× *g*, for 40 min at 4 °C. The pellets were stored at −80 °C until use. The bacterial pellets were thawed and resuspended in lysis buffer (20 mM phosphate buffer, pH 7.4), supplemented with protease inhibitor cocktail (#P8340, Sigma-Aldrich). For 10 g of pellet, 100 mL of lysis buffer was added. The cells were lysed by a French pressure cell press (AMINCO, American Instrument Co., Silver Spring, MD, USA). To remove cell debris, the cell lysates were centrifuged for 45 min at 26,200× *g*. The supernatant was adjusted to pH 7.5 and diluted with ddH_2_O until a conductivity of 4 mS/cm was reached and matched with the DE52 cellulose anion-exchange column (Whatman, Piscataway, NJ, USA). Adjusted supernatant was passed through the equilibrated DE52 column. Most of the bacterial proteins were bound to the column, and Cyt*c* was collected in the flow-through. The collected solution was adjusted to pH 6.5 and conductivity was increased by the addition of KH_2_PO_4_ buffer until it reached a conductivity of 6 mS/cm and matched with the CM52 cellulose cation-exchange column (Whatman). The adjusted flow-through was passed through the equilibrated CM52 column, allowing the positively charged Cyt*c* to bind the CM52 column. After washing the CM52 column, the bound Cyt*c* was eluted with high salt buffer (0.5 M NaCl in 40 mM phosphate buffer, pH 6.5). Cyt*c* fractions were desalted and concentrated by centrifugation using Amicon ultra-15 3 k units (#UFC901008, Millipore, Billerica, MA, USA). The purity of the protein was determined by spectrophotometer and by running 1 µg of each Cyt*c* variant on a 10% Tris-tricine gel, followed by Coomassie blue staining.

### 2.5. Determination of the Concentration of Cytc Variants

Diluted Cyt*c* variants were oxidized with a few granules of potassium ferricyanide K_3_Fe(CN)_6_ and reduced with a few granules of sodium dithionite (Na_2_S_2_O_4_). Absorption spectra were recorded on a Jasco V-570 double beam spectrophotometer (2 nm bandwidth, 200 nm/min scanning speed) using a 0.1 mm path length quartz cuvette. In order to determine concentration, the absorbance at 550 nm was recorded for reduced and oxidized Cyt*c*. The Cyt*c* concentration in mM was calculated using the following equation: (A550_red_−A550_oxi_)/19.6 mM/cm × 1 cm × dilution factor.

### 2.6. COX Activity Measurement

Regulatory-competent bovine liver COX (3 µM) was dialyzed at 4 °C in the presence of cardiolipin and 0.1 mM ATP in COX measuring buffer (10 mM K-HEPES pH 7.4, 40 mM KCl, 2 mM EGTA, 10 mM KF, 1% Tween-20), as previously described [11], to remove cholate bound to COX during enzyme purification. Oxygen consumption of COX (30 nM) upon titration of Cyt*c* variants (0–25 µM) was analyzed using a Clark-type oxygen electrode (Oxygraph system, Hansatech, Pentney, UK) at 25 °C in 220 µL of COX measuring buffer in the presence of 20 mM ascorbate as the electron donor. The oxygen consumption rate was recorded and analyzed with the Oxygraph software (Hansatech). The activity of COX was expressed as turnover number (s^−1^).

### 2.7. Caspase-3 Activity Measurement

Caspase-3 activity was assayed using an in vitro cell-free apoptosis detection system with cytosolic extracts from HeLa cells as previously described [19,20]. Cells were cultured in eight 75 cm^2^ flasks, harvested by trypsinization, and washed twice with cold PBS, followed by one wash with cytosolic extraction buffer (CEB: 20 mM K-HEPES, pH 7.5, 1.5 mM MgCl_2_, 10 mM KCl, 1 mM EGTA, 1 mM EDTA, 1 mM DTT, 100 μM PMSF). The cell pellet was resuspended in CEB, and the suspension was transferred to a Dounce homogenizer, allowing it to swell in the hypotonic CEB for 15 min on ice. Cells were disrupted by 20 strokes with a B-type glass pestle. Lysates were centrifuged at 15,000× *g* for 15 min at 4 °C to collect the cytosolic extract. Protein concentration was determined using the DC protein assay kit (Bio-Rad, Hercules, CA, USA). Caspase-3 activity was measured using the EnzChek caspase-3 assay kit (Invitrogen, Carlsbad, CA, USA). Artificial caspase-3 substrate rhodamine 110-linked DEVD tetrapeptide (Z-DEVD R110), which fluoresces upon cleavage by caspase-3, was used to determine enzyme activity. Cytosolic extracts at a protein concentration of 2 mg/mL were incubated with recombinant K53 Cyt*c* variants (15 μg/mL) along with 1 mM ATP for 2.5 h at 37 °C. Caspase-3 inhibitor (Ac-DEVD-CHO) was used as a negative control. Fluorescence was measured using a Fluoroskan Ascent FL plate reader (Labsystems, Thermo Fischer Scientific) as previously described [12]. Fluorescence readings from cytosolic extracts without Cyt*c* were subtracted as a background. Data were presented as a % of change compared to WT.

### 2.8. Rate of Oxidation Measurement

Rate of oxidation was measured as previously described [11,21]. Briefly, Cyt*c* variants were reduced with Na_2_S_2_O_4_, and the proteins were separated from the reductant by passing through NAP-5 columns (#17-0853-02, GE Healthcare, Piscataway, NJ, USA). Oxidizing agent H_2_O_2_ (50 µM) was added to Cyt*c* (15 µM) in 0.2 M Tris-Cl, pH 7.0. After 10 s of H_2_O_2_ addition, the decrease of the absorption peak at 550 nm was measured. The amount of oxidized Cyt*c* was calculated as described above to calculate the rate of Cyt*c* oxidation (s^−1^).

### 2.9. Rate of Reduction by Superoxide

The hypoxanthine/xanthine oxidase reaction system was used to generate superoxide in a cuvette as described [22,23] with some modifications. K53 Cyt*c* variants were oxidized with K_3_Fe(CN)_6_ and desalted on a NAP-5 column (GE Healthcare). The reaction consisted of 4 µM ferri-Cyt*c,* 100 µM hypoxanthine, and 14.2 nM catalase in 1X PBS. After obtaining the initial spectrophotometric reading at 550 nm, 181.5 nM xanthine oxidase was quickly added to the cuvette to initiate the reaction. The 550 nm absorbance was measured at 30 s. The concentration of reduced Cyt*c* was calculated as described above and the rate of Cyt*c* reduction by superoxide was determined. Superoxide dismutase 2 (925 nM) was used as a negative control that rapidly converts superoxide into H_2_O_2_. All the reagents were purchased from Sigma unless otherwise stated.

### 2.10. Cardiolipin Peroxidase Activity Measurement

The cardiolipin peroxidase activity of Cyt*c* was measured as previously described [11] with modifications. Liposomes containing 0%, 20%, 30%, and 50% of 18:1 tetraoleoyl-cardiolipin (TOCL) and 1,2-dioleoyl-sn-glycero-3-phosphocholine (DOPC) were generated. Lyophilized DOPC was solubilized in chloroform to prepare a working stock of 10 µg/µL. The required volume of solubilized DOPC for each liposome was added to the tubes containing TOCL solubilized in chloroform, and both lipids were evaporated under nitrogen to remove the solvent. Lipids were resuspended in 20 mM K-Hepes buffer, pH 7.2, and liposomes were prepared by sonication for 30 s on ice, 5 times, with 1 min intervals. Liposomes (25 µM) were incubated in a 96-well plate with 1 µM Cyt*c* in the presence of 20 mM K-Hepes. After addition of Cyt*c,* the plate was incubated at room temperature for 10 min to allow the association and equilibration of Cyt*c*-cardiolipin binding. Fluorescence of resorufin, the oxidation product of Amplex red, was detected using a Fluoroskan Ascent microplate reader (Labsystems, Thermo Fisher Scientific) with excitation and emission wavelengths of 530 nm and 590 nm, respectively. The reaction was started with the addition of 10 µM Amplex Red and 5 µM H_2_O_2_ using a multichannel pipette. The reaction progress was monitored for 5 min, during which the reaction rate was linear. Fluorescence over the 5 min time span was reported as fluorescence (AU) per min.

### 2.11. Redox Potential Measurement

The midpoint redox potential (E^0′^) was analyzed spectrophotometrically by the equilibration method [24] using 2,6-dichloroindophenol (DCIP, E^0′^ = 237 mV) as a reference compound, which has an absorption band at 600 nm in its oxidized state. One hundred μL of Cyt*c* solution (2 mg/mL) were mixed in a spectrophotometric cuvette with 200 μL of 50 mM citrate buffer (pH 6.5), 10 μL of 1 mM DCIP, and 2.5 μL of 1 mM K_3_Fe(CN)_6_ to fully oxidize Cyt*c*. Absorbance values corresponding to fully oxidized Cyt*c* (A550-A570) and DCIP (A600) were recorded using a Jasco V-570 double beam spectrophotometer. The mixture was then sequentially reduced by adding 0.5 μL of 1 mM ascorbate (pH 6.5), and absorbance values were acquired at each step. When readings became constant, a few grains of Na_2_S_2_O_4_ were added to fully reduce Cyt*c* and DCIP. For each step, ratios of oxidized and reduced forms of both compounds were calculated. Data obtained were plotted as log(DCIP_OX_/DCIP_RED_) versus log(Cyt*c*_OX_/Cyt*c*_RED_), yielding a linear graph with a slope of n_DCIP_ /n_Cyt*c*_ and a *y*-axis intercept of n_Cyt*c*_/59.2 (E_Cyt*c*_ − E_DCIP_) as previously described [19]. These values were used to calculate the E^0′^ (mV) of Cyt*c* from the Nernst equation.

### 2.12. Crystallization of K53Q Cytc

Cyt*c* variants were further purified by gel filtration on a Sephacryl S-100 column (GE healthcare). The protein was oxidized with 5 mM K_3_Fe(CN)_6_ to establish a defined oxidation state before crystallization. Crystallization was done using basic crystallization screens (Jena bioscience, Jena, Germany) with an approximate protein concentration of 15–20 mg/mL. Crystals were grown by vapor diffusion after mixing 1 µL of protein solution with 1 µL of the reservoir solution and equilibrating the drop against 0.5 mL of the precipitant at room temperature. The best diffracting crystals of K53Q mutant grew to 0.3 × 0.1 mm size in JBS1D4 condition which was 30% PEG 2K MME, 0.1 M MES 6.5, and 0.1 M Na-acetate. Crystals were soaked for 10 min in a cryoprotectant solution (20% *v*/*v* ethylene glycol) before flash freezing in liquid nitrogen. Single crystal diffraction data were collected at the Life Sciences Collaborative Access Team beamline 21-ID-D at the Advanced Photon Source, Argonne National Laboratory. The data were collected over a full 360° rotation in either 0.6 or 1.0 frames and integrated using XDS [25] in AutoProc [26].

### 2.13. Statistical Analyses

Experimental data consisting of 3 or more replicates were analyzed for statistical significance for the different experiments by one-way ANOVA followed by post hoc Tukey test using GraphPad Prism version 8.4.3 (GraphPad software, San Diego, CA, USA). Data are reported as means ± SEM and were considered statistically significant (*) with *p* < 0.05.

### 2.14. Molecular Dynamics

Molecular dynamics simulations were performed with YASARA version 20-07-04 [27] using the conservative “slow” protocol and the recommended default forcefield, AMBER 2014 [28]. The starting structure for the molecular dynamics calculations on WT is from molecule A in 5C0Z.PDB, which was obtained from oxidized Cyt*c* in the presence of potassium ferricyanide (K_3_[Fe(CN)_6_]), and the K53 mutants were all modeled from WT molecule A. Root mean square fluctuation (RMSF) plots for the amino acid side chains were generated with GraphPad using data imported from YASARA.

## 3. Results

### 3.1. Cytc Is Acetylated on Lysine 53 in Castrate-Resistant and Castrate-Sensitive Human Prostate Cancer Xenografts

**Figure 1:**
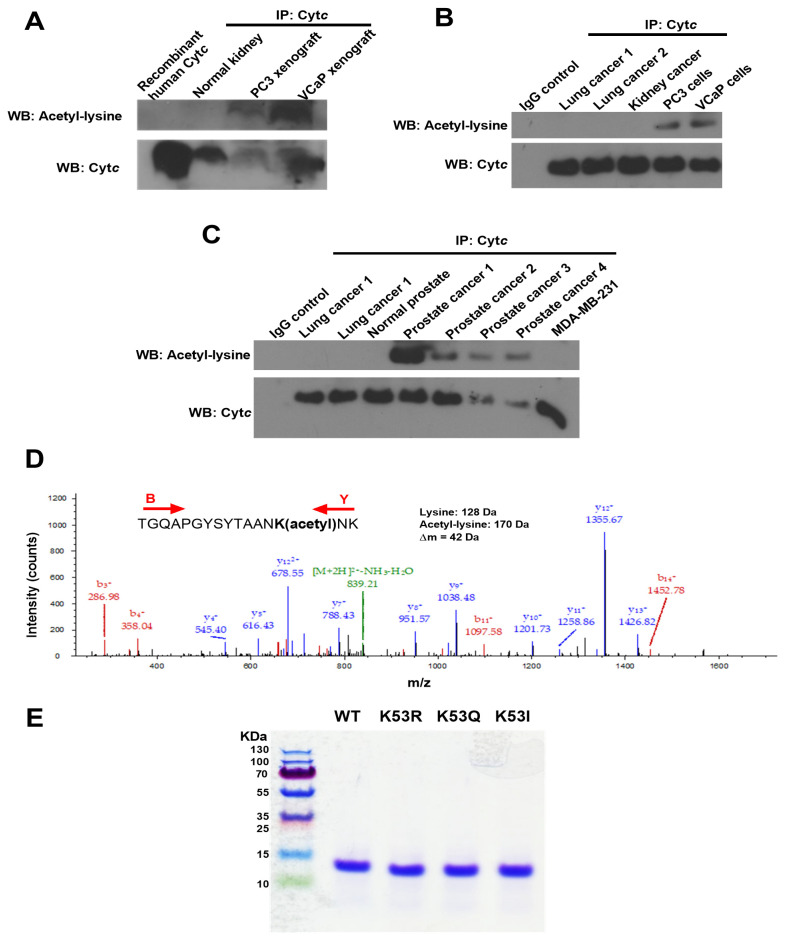
(**A**) Cyt*c* is acetylated in castrate-resistant (PC3) and castrate-sensitive (VCaP) prostate cancer xenograft tissues compared to normal human kidney tissue and recombinant human Cyt*c*. (**B**) Cyt*c* is acetylated in cultured PC3 and VCaP cells, but not acetylated in lung and kidney cancer tissues. (**C**) Cyt*c* is acetylated in human prostate cancer tissues, but not acetylated in normal prostate tissue, lung cancer tissue, and MDA-MB-231 cells. (**D**) Mass spectrum of Cyt*c* peptide TGQAPGYSYTAANK(acetyl)NK identifying Cyt*c* K53 acetylation (underlined). This spectrum represents one immunoprecipitated castrate-sensitive tumor xenograft. The acetyl moiety was unambiguously assigned to this lysine via fragment ions y4+, y5+, etc., and b14+ containing 42 extra mass units for the acetyl group. The peptide sequence was unambiguously revealed by fragment ions b3+, b4+, b11+, b14+, y4+, y5+, y7+, y8+, y9+, y10+, y11+, y12+, and y13+. (**E**) Coomassie blue-stained gel showing the purity of recombinant Cyt*c* overexpressed and purified from bacteria. All 4 Cyt*c* variants displayed a single band on a 10% tris-tricine SDS-polyacrylamide gel at around 12 kDa.

This is the first study to explore the role of Cyt*c* PTMs in the context of cancer. As there can be a time gap from the initial tumor resection or biopsy until pathological examination and cryopreservation of the clinical specimen, which can lead to the loss of PTMs or their artificial introduction, we here chose the xenograft model. We implanted castrate-resistant (PC3) and castrate-sensitive (VCaP) human prostate cancer cells in SCID mice and harvested the xenografts when they reached a mass of about 800 mg. Samples were removed within 1 min and stored at −80 °C until use. As the available tissue amount is small, a biochemical purification was not possible, and we instead enriched Cyt*c* by immunoprecipitation. Western blot analysis showed that Cyt*c* from prostate cancer xenografts are acetylated compared to recombinant human Cyt*c* and Cyt*c* immunoprecipitated from normal human kidney tissue (Figure 1A). Cyt*c* was also acetylated in cultured PC3 and VCaP cells (Figure 1B). Furthermore, Cyt*c* was acetylated in prostate cancer tissues, whereas normal prostate tissue, lung cancer tissue, and MDA-MB-231 breast cancer cells did not indicate acetylation (Figure 1C). Next we performed mass spectrometry on Cyt*c* immunoprecipitated from a total of 8 prostate cancer xenografts (4 castrate-resistant and 4 castrate-sensitive). We found that K53 of Cyt*c* was acetylated in all 8 independently grown prostate cancer xenografts (Figure 1D). Human Cyt*c* has 18 lysine residues, however, only K53 was found to be acetylated in these samples suggesting that K53 acetylation is a highly specific modification.

### 3.2. Purification of WT, Acetylmimetic, and Additional Cytc Control Variants

As the cost to grow enough xenografts to obtain preparative amounts of Cyt*c* for functional studies is prohibitive, we replaced the lysine residue with glutamine, which mimics acetylated lysine. This mutation (K53Q) converts the positive charge of the lysine side chain to a neutral charge, similar to the effect of lysine acetylation seen with the acetylated protein in vivo. The bacterial pLW01 plasmid was used to generate WT and acetylmimetic K53Q Cyt*c*. We included two additional control mutants, K53R Cyt*c*, which carries a positive charge similar to non-acetylated lysine and nonpolar K53I. While K53 is evolutionarily conserved across all mammals, K53I is the next most common replacement at this site among species such as yeast and drosophila [29]. Recombinant WT, K53Q, K53R, and K53I Cyt*c* were overexpressed in bacteria and purified to homogeneity. The purified proteins were resolved on an SDS-polyacrylamide gel and stained with Coomassie blue (Figure 1E). All 4 proteins resulted in a single band around 12 kDa confirming the purity of Cyt*c* used for the functional assays.

### 3.3. K53Q Acetylmimetic Cytc Results in Reduced Cytochrome c Oxidase and Caspase 3 Activity Compared to WT

**Figure 2:**
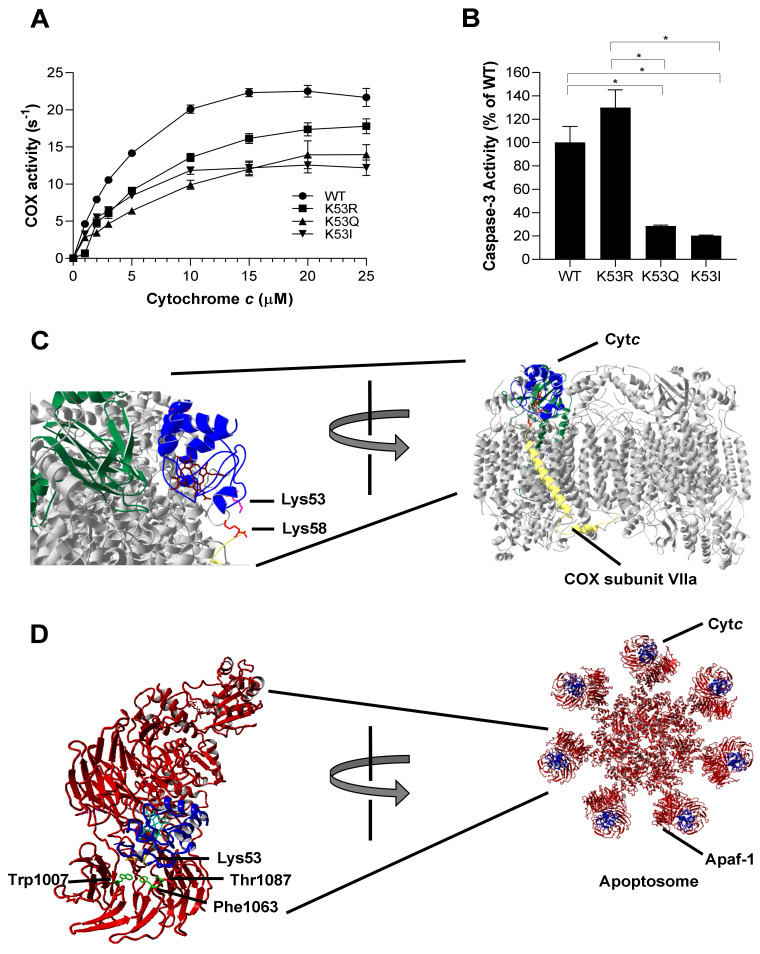
(**A**) Oxygen consumption rate was measured in the presence of 30 nM of bovine liver COX using an Oxygraph system. WT, K53R, K53Q, and K53I Cyt*c* variants were titrated at concentrations of 0, 1, 2, 3, 5, 10, 15, 20, and 25 µM (*n* = 3). Data are represented as means±SEM. (**B**) Cytosolic extracts of HeLa cells were incubated with recombinant WT, K53R, K53Q, and K53I variants for 2.5 h at 37 °C. Fluorescence that resulted from caspase-3 mediated cleavage of the rhodamine substrate DEVD-R110 was used as a measure of caspase-3 activity (*n* = 3). Data are represented as means ± SEM, * *p* < 0.05. (**C**) Docking model of COX and Cyt*c* [32] showing the interaction of Lys53 residue of Cyt*c* with Lys58 residue of COX subunit VIIa within 5 Å. (**D**) Cyt*c* Lys53 interaction with Apaf-1 represented on the human apoptosome structure [33]. Apaf-1 residues Phe1063 (within 5 Å) and Trp1007 and Thr1087 (within 6 Å) are in close proximity to Lys53 residue.

Cancer metabolism is often characterized by increased glycolysis and decreased mitochondrial respiration, leading to the Warburg effect [30]. To analyze the role of K53 acetylation in respiration, purified K53 Cyt*c* variants were used to determine oxygen consumption rate in the reaction with COX. Regulatory-competent COX was isolated from bovine liver under conditions that preserve the in vivo phosphorylation state [31]. Oxygen consumption of 30 nM bovine liver COX was measured via titration (1–25 µM) of purified Cyt*c* variants in an Oxygraph system using a Clark-type oxygen electrode. The experimental results showed that acetylmimetic K53Q and K53I Cyt*c* resulted in a lower oxygen consumption rate compared to WT and K53R Cyt*c* (Figure 2A). At maximal turnover, COX activity was reduced by about 35% in the presence of K53Q acetylmimetic Cyt*c* compared to the WT. The data suggest that K53 acetylation of Cyt*c* downregulates OxPhos, thus supporting Warburg metabolism. We studied the interaction of K53 Cyt*c* with COX using the published docking model [32] of COX and Cyt*c* and found that K53 is within 5 Å of lysine 58 residue of COX subunit VIIa (Figure 2C). This model suggests that K53 is within the COX binding domain of Cyt*c* and altering this residue or adding an acetyl group could spatially interfere with optimal binding, resulting in decreased COX activity.

Cancer cells often manage to bypass cell death pathways, and evasion of apoptosis is therefore another hallmark of cancer [8]. We analyzed the ability of K53 Cyt*c* variants to initiate apoptosis by measuring downstream caspase-3 activity. WT, K53Q, K53R, and K53I recombinant Cyt*c* proteins were incubated with cytosolic extracts isolated from HeLa cells. Caspase-3 activity was determined by rhodamine fluorescence upon cleavage of the rhodamine-linked DEVD tetrapeptide. Interestingly, acetylmimetic K53Q and nonpolar K53I resulted in significantly 80% and 87% reduced caspase-3 activity, compared to the WT (Figure 2B). Our control K53R Cyt*c* even showed slightly increased caspase-3 activity, suggesting that a positive charge is required for maximal caspase activation. A closer analysis of the human apoptosome structure [33] shows that K53 is a part of the Apaf-1 binding domain of Cyt*c* (Figure 2D). This further confirms that K53 serves as a regulatory epitope for Apaf-1 binding and the activation of the caspase cascade and suggests an underlying mechanism for evasion of apoptosis in prostate cancer through K53 acetylation.

### 3.4. Acetylmimetic Cytc Is a Superior ROS Scavenger

**Figure 3:**
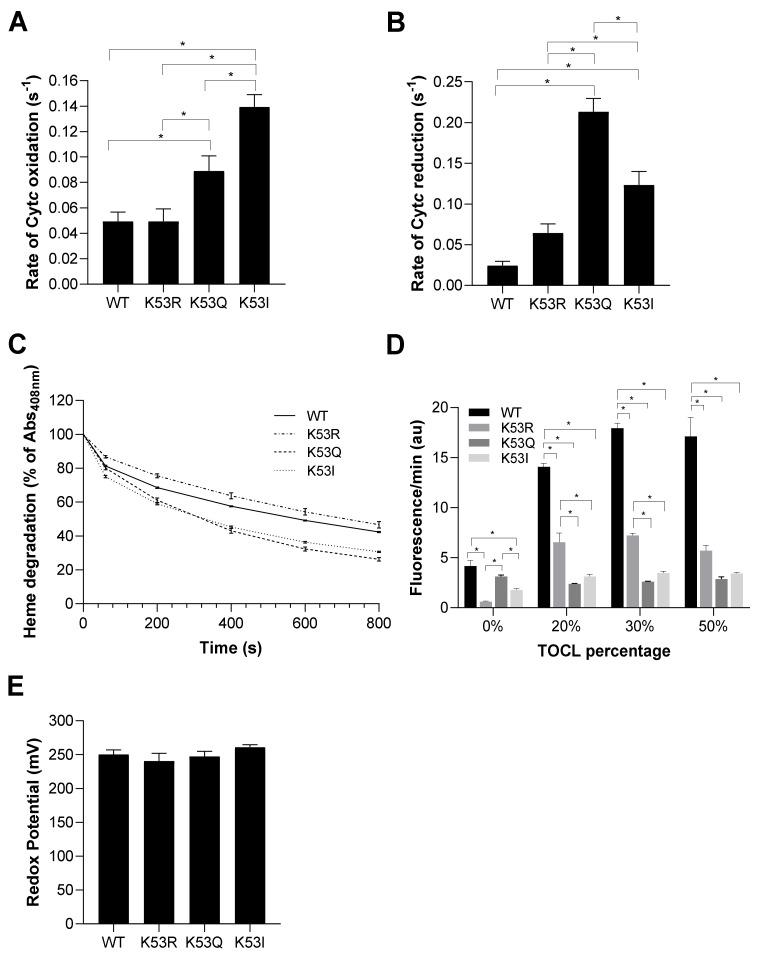
(**A**) Rate of oxidation of reduced Cyt*c* 10 s after the addition of 50 µM H_2_O_2_ (*n* = 7). (**B**) Rate of reduction of oxidized Cyt*c* by superoxide produced in the presence of the hypoxanthine/xanthine oxidase reaction (*n* = 4–8). (**C**) Heme degradation of Cyt*c* upon addition of excessive H_2_O_2_ (3 mM) was measured as a reduction of absorbance in the 408 nm Soret peak of Cyt*c* (*n* = 3). (**D**) Liposomes containing 0%, 20%, 30%, and 50% of tetraoleoyl-cardiolipin (TOCL) were incubated in the presence of Cyt*c* K53 variants. Cardiolipin peroxidase activity was determined in the presence of H_2_O_2_ and Cyt*c* as a measure of resorufin fluorescence (*n* = 5). (**E**) Redox potential of K53 variants of Cyt*c* were measured using the spectrophotometric method in the presence DCIP as a reference compound (*n* = 5). Data are represented as means ± SEM, * *p* < 0.05.

Cyt*c* functions as a scavenger for both hydrogen peroxide and superoxide [34,35]. Considering the antioxidant role of Cyt*c*, we determined the rate of Cyt*c* oxidation upon hydrogen peroxide addition and the rate of Cyt*c* reduction upon superoxide production. To determine the rate of Cyt*c* oxidation, Cyt*c* variants were reduced with sodium dithionite and the ferro-Cyt*c* variants were oxidized in the presence of 50 µM H_2_O_2_. K53Q undergoes oxidation at about twice the rate compared to the WT and K53R Cyt*c* controls, while K53I mutant resulted in a 2.8-fold higher rate (Figure 3A). We next used the xanthine oxidase/hypoxanthine system to generate superoxide [36] to analyze the superoxide scavenging capacity of recombinant Cyt*c* variants. Cyt*c* was fully oxidized and reduction of Cyt*c* through electron transfer from superoxide was determined by spectrophotometer. Interestingly, K53Q and K53I Cyt*c* resulted in a 9- and 5-fold increase in superoxide scavenging activity compared to WT Cyt*c* (Figure 3B). These functional changes could add to the pro-survival features of Cyt*c* in prostate cancer by detoxifying ROS and lowering apoptosis, as ROS are a major apoptotic trigger.

### 3.5. Acetylmimetic Cytc Is Less Stable at High H_2_O_2_ Concentrations and Shows Profoundly Reduced Cardiolipin Peroxidase Activity

Cyt*c* can lose its functionality at very high ROS concentrations. To test its stability, we measured degradation of the heme catalytic site of Cyt*c* upon addition of a high concentration (3 mM) of H_2_O_2_. Dissipation of the Soret peak of Cyt*c* at 408 nm is characteristic of heme degradation. K53Q replacement resulted in the highest heme degradation of 73.8% at 800 s compared to WT, K53R, and K53I that resulted in a heme degradation of 57.6%, 53.3%, and 69.3%, respectively (Figure 3C). Cardiolipin is a mitochondria-specific phospholipid which interacts with Cyt*c*. The peroxidase activity of Cyt*c* is activated in the presence of H_2_O_2_, resulting in cardiolipin peroxidation. If this reaction happens on the outer mitochondrial membrane, Cyt*c* can be released from the mitochondria leading to activation of apoptosis [3,4]. Cardiolipin peroxidase activity of Cyt*c* variants was analyzed by their efficiency in oxidizing cardiolipin, which in turn oxidizes Amplex red to resorufin. Interestingly, cardiolipin peroxidase activity of acetylmimetic K53Q and nonpolar K53I Cyt*c* mutants were significantly reduced, by between 78% and 86%, compared to WT Cyt*c* at TOCL percentages of 20%, 30%, and 50% (Figure 3D). These findings align with caspase-3 activity data, further supporting an anti-apoptotic role for K53 acetylation. Finally, for proper functioning of Cyt*c* as an electron carrier, the redox potential of Cyt*c* should be between the redox potentials of complexes III and IV. In the literature, the mammalian Cyt*c* redox potentials are in the range of 220–270 mV [24,37]. The midpoint redox potential (E^0′^) of K53 Cyt*c* variants was measured spectrophotometrically. The redox potentials of the Cyt*c* variants were similar, 250 mV, 241 mV, 247 mV, and 261 mV for WT, K53R, K53Q, and K53I Cyt*c*, respectively (Figure 3E), suggesting that modification of K53 does not have a major effect on the heme moiety.

### 3.6. Crystal Structure of K53Q Cytc at a Resolution of 1.31 Å

**Figure 4:**
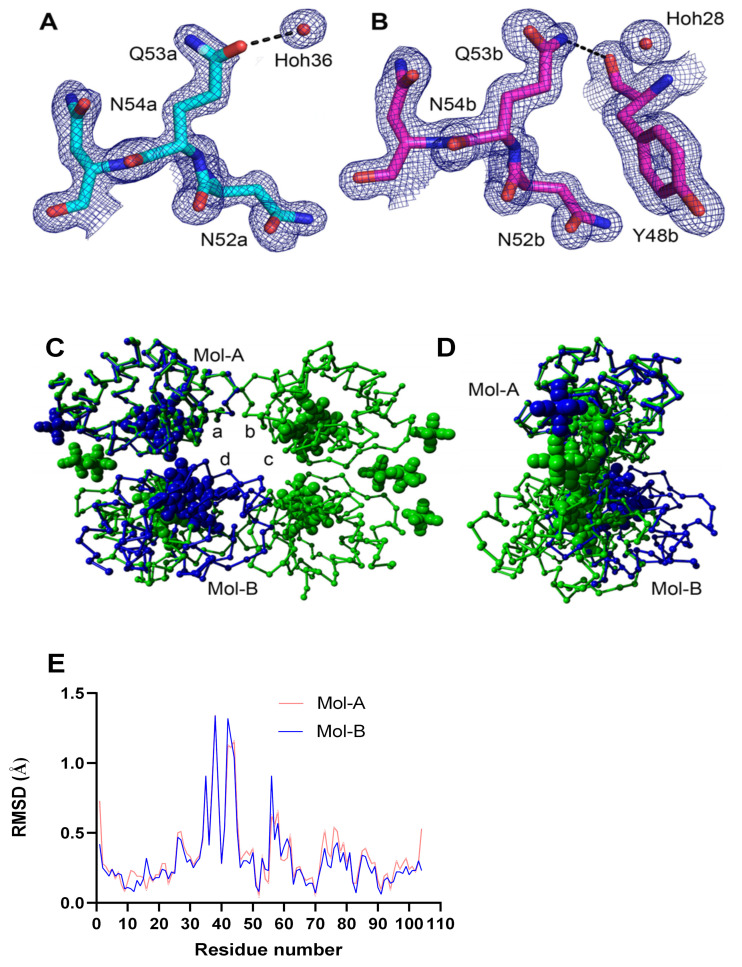
(**A**,**B**) The comparison of the “omit” density for K53Q in the two Cyt*c* molecules in the asymmetric unit of the crystal structure. (**C**,**D**) The structure of the K53Q Cyt*c* crystallographic dimer is superposed onto the native rodent Cyt*c* (5C0Z.pdb) crystallographic tetramer using chain-A in both structures. (**E**) C-alpha atom RMSD plots of K53Q Mol-A and Mol-B relative to Mol-A in the WT structure (5C0Z.pdb).

We have previously successfully crystalized WT and phosphomimetic Cyt*c* variants [11,13] to explore possible structural changes and to draw structure and function relationships. We used the same approach here and attempted to crystalize all three Cyt*c* mutants. Only the K53Q acetylmimetic mutant crystallized and gave a high-resolution structure at 1.31 Å in space group P2_1_2_1_2 with two independent monomers in the asymmetric unit. Structural data have been posted under the PDB code 7LJX. The crystallographic parameters for the K53Q structure are compared to those of the native mouse Cyt*c* (5C0Z.pdb) and the Y46F human Cyt*c* (3ZOO.pdb) structures in Table 1. The components in the crystal structure of the K53Q mutant that are within 3.5 Å of Q53 in molecules A and B are presented (Figure 4A,B). The electron density for the two K53Q residues from an unbiased “omit” map is shown at 1.0 sigma. The electron density for Q53 in molecule B is better defined because its side chain is immobilized by a hydrogen bond to the carbonyl oxygen of Y40. The side chain of Q53 in molecule A interacts only with a water molecule within 3.5 Å. The crystal structure of K53Q Cyt*c* (blue) was superposed onto the structure of native (5C0Z.pdb) Cyt*c* (green) using only the A-chains in both structures (Figure 4C,D). The four independent chains in the native structure are labeled with lower case letters. The two independent chains in the K53Q Cyt*c* structure are labeled with capital letters. The RMSD value for the superposed A-chains was 0.423 Å calculated from the backbone atoms. The protein structures are depicted as Cα traces with the atoms in the hemes and the ferrihexacyanide molecules represented by spheres. Figure 4C shows the front face of the 5C0Z tetramer in the P1 asymmetric unit. Figure 4D shows the edge of the tetramer after a 90° rotation about the vertical axis. When the two structures are superposed using only the A-chains, the B-chain in the K53Q Cyt*c* structure partially overlaps with the D-chain in 5C0Z, but their relative orientation in the two crystal structures differs dramatically (RMSD = 20.3 Å).

The RMSD plot comparing the two independent K53Q molecules (7LJX.pdb) with molecule A in the native Cyt*c* structure (5C0Z.pdb) shows a significant difference—exhibited by both K53Q molecules—at residues R38 and Q42 (Figure 4E). These two residues plus the three intervening residues have a total of 4 contacts within 4 Å with two adjacent dimers in the crystal. One of the crystal interfaces is reciprocal—K39 in the central dimer is within 4Å of K39 in the adjacent dimer.

### 3.7. Molecular Dynamics Simulations

**Figure 5:**
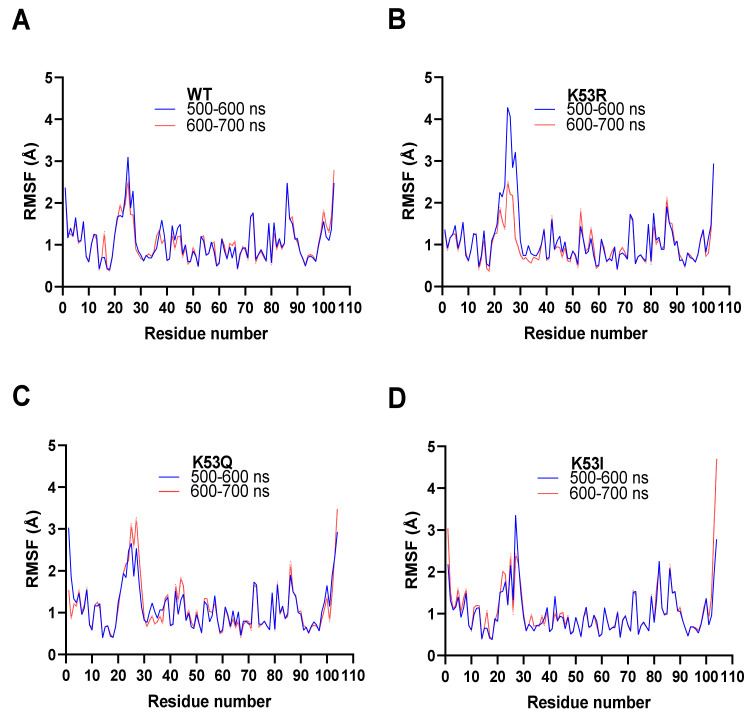
Molecular dynamics simulations. All four simulation averages were calculated for the 500–600 ns (blue) and 600–700 ns (red) intervals. The average RMSF (Å) for all atoms in each residue in the amino acid chains are shown. All four runs displayed equilibrium within 500 ns to 700 ns. (**A**) Molecule A from the WT Cyt*c* crystal structure (PDB entry 5C0Z). (**B**) Equivalent to (**A**) for K53R Cyt*c*. (**C**) Equivalent to (**A**) for K53Q Cyt*c*. (**D**) Equivalent to (**A**) for K53I Cyt*c*.

Molecular dynamics calculations were performed on four Cyt*c* variants (WT, K53R, K53Q, and K53I) using YASARA version 20-07-04 with its conservative “slow” protocol and the default forcefield, AMBER 2014. Each dynamics calculation was run for 700 ns—the same as our recent study on the phosphomimetic, T58E Cyt*c* [12]. The residue RMSF values for the 600 ns and 700 ns blocks for WT and the three mutants were compared in this study (Figure 5). The two blocks are similar for WT, K53Q, and K53I, which indicates that the calculations have reached equilibrium. The K53R model has significantly less mobility in the loop consisting of amino acid residues 20–30 at 700 ns than at 600 ns. All four plots in Figure 5 show significant mobility (>2.5 Å) between residues 20–30 and at the C-terminus. The other site with RMSF values approaching 2 Å consist of residues 81–90. Met80 is tethered to the heme iron atom. Similar patterns were observed for the T58 mutants [12]. Although Figure 4E shows significant RMSD differences between the WT and K53Q crystal structures at residues 38 and 42, the RMSF values are relatively small (Figure 5A,C).

## 4. Discussion

In this study, we discovered that K53 acetylation of Cyt*c* is a prostate cancer specific modification that may contribute to the underlying mechanisms of cancer. This modification was present in both castrate-sensitive and castrate-resistant tumor xenografts, suggesting that K53 acetylation of Cyt*c* plays a role in prostate cancer pathogenesis regardless of hormone sensitivity of the tumor. Cyt*c* is a highly positively charged protein that has 18 lysine residues which account for over one-sixth of the protein. Lysine acetylation is the most common PTM of the mitochondria, and in particular in the matrix [38,39]. The pumping of protons across the inner mitochondrial membrane (IMM) to generate the proton-motive force results in a more basic pH between 7.9 and 8.0 in the mitochondrial matrix, compared to other compartments such as the cytosol and nucleus (pH 7.2) [40]. The basic pH and the abundance of acetyl-CoA creates a more favorable environment for non-enzymatic protein lysine acetylation in the mitochondrial matrix [41,42].

However, Cyt*c* is located in the IMS with a more acidic pH compared to the matrix. Strikingly, K53 was consistently acetylated in all 8 prostate cancer xenograft samples subjected to mass spectrometry analysis, making it a highly specific modification. Cyt*c* immunoprecipitated from prostate cancer and prostate cancer cell lines was also acetylated, whereas Cyt*c* from normal prostate, lung cancer, normal and kidney cancer tissues, and a breast cancer cell line was not acetylated. It should also be noted that phosphorylations, which we have reported in healthy tissues and have been shown to regulate the multiple functions of the protein [9,10,11,12,13,43,44,45,46,47,48], were not detected in any of the prostate tumor xenografts. This indicates a dichotomy between signaling pathways in normal and transformed cells that target Cyt*c*. Additionally, K53 of Cyt*c* is conserved across all mammals [29], pointing to a central regulatory site.

Cell signaling by PTMs is one of the most common modes of regulation in higher organisms that is dysregulated in cancer. The two hallmarks of cancer, metabolic reprogramming and evasion of apoptosis, could at least in part be explained by Cyt*c* acetylation. Cancer cells undergo metabolic reprogramming that often results in reduced OxPhos activity, which together with parallel upregulation of glycolysis allows fast ATP production and provides metabolites for biosynthesis for cancer cell proliferation [7,49,50]. Cyt*c* is also a central player in apoptosis and has several other functions such as ROS scavenging, which could be exploited by cancer cells. Therefore, in order to characterize K53 acetylation, we used an acetylmimetic substitution, K53Q. This is a well-established method for studying acetylation in vitro [51]. The advantages of this approach are that the modification cannot be lost during functional assays and that large amounts of the protein can be generated by bacterial overexpression for functional analyses. In addition to K53Q, we assessed the function of WT, K53R, and K53I variants of Cyt*c*. WT and K53R carry a positive charge at residue 53, whereas K53Q and K53I are polar and neutral, respectively, but do not carry a charge. Consequently, in most assays, K53Q and K53I behaved in a similar manner.

This reaction between Cyt*c* and COX is the proposed rate-limiting step of the ETC under physiological conditions where 90% of cellular oxygen is consumed [52]. K53Q acetylmimetic replacement resulted in significantly lower oxygen consumption in reaction with COX compared to WT and K53R. Therefore, our results suggest that K53 acetylation plays a role in metabolic reprogramming by lowering ETC activity. Our results further suggest that K53 acetylation of Cyt*c* may lead to evasion of apoptosis by hindering apoptosome formation and activation. Interestingly, additional pro-survival effects of K53Q acetylmimetic replacement are increased ROS scavenging and decreased cardiolipin peroxidase activity. Oxidative stress mediated by ROS such as superoxide and H_2_O_2_ are considered triggers of apoptosis [53]. K53Q Cyt*c* showed increased ability to scavenge both superoxide and H_2_O_2_. However, the role of ROS in cancer is complicated and cancer stage specific. In certain stages increased ROS levels can also promote cancer as, for example, has been shown for ROS scavenger manganese superoxide dismutase (MnSOD), which can act both as a tumor suppressor and a promoter [54]. Cardiolipin peroxidase activity is also classified as a pro-apoptotic function [55]. Lower cardiolipin peroxidase activity in the presence of K53Q Cyt*c* suggests that less Cyt*c* is available to be released from the mitochondria into the cytosol to activate apoptosis when Cyt*c* is acetylated at K53.

Of the new mutants studied here only the K53Q Cyt*c* mutant crystallized and gave a high-resolution structure at 1.31 Å that showed good omit map density for Q53 and confirmed that the mutation caused no major structural disturbance or unfolding at its location, although there is a moderate distortion at R38-Q42 due to crystal packing. Interestingly, molecular dynamics simulations revealed that K53Q has increased flexibility at the T28 epitope, a loop previously shown to be an important regulatory domain [11]. We propose that the increased mobility of this loop may explain the increased ROS scavenging activities of acetylmimetic Cyt*c* due to a more flexible and thus open structure, allowing easier access of superoxide and H_2_O_2_ to the heme group. We also showed that K53Q Cyt*c* undergoes faster heme degradation in the presence of excessive H_2_O_2_, which is consistent with our interpretation of better access of the oxidant to the heme group. A previous study with T28A Cyt*c* also showed a similar pattern both functionally and in molecular dynamics simulations [11], assigning an important regulatory and stability function to the T28 loop.

In conclusion, our studies suggest that K53 acetylation is a pro-cancer modification by lowering COX activity and caspase-3 activity and by increasing ROS scavenging. To our knowledge, this is the first report characterizing a specific Cyt*c* acetylation. We propose that K53 acetylation of Cyt*c* contributes to cancer pathogenesis by promoting Warburg metabolism and evasion from apoptosis. However, these mechanisms should be further confirmed in a cell culture system and in vivo. Furthermore, there is an ongoing debate with regard to OxPhos activity in prostate cancer. Some studies suggest an increase in OxPhos activity [56,57,58] while others suggest a decrease in mitochondrial respiration with increasing malignancy [59,60,61]. Therefore, more emphasis should be given to the anti-apoptotic role of Cyt*c* K53 modification in prostate cancer cells. A recent study showed that abrogation of apoptosome-mediated caspase activation and defective release of Cyt*c* from the mitochondria are associated with prostate cancer aggressiveness observed in African American men [62], further supporting an important role of Cyt*c* as a cancer driver. Other studies have shown that acetyltransferases mediating protein acetylation are upregulated in prostate cancer [63,64] and could be established as rational therapeutic targets [65,66]. Therefore, a better mechanistic understanding of Cyt*c* acetylation in prostate cancer and potentially other cancers may allow the development of novel effective cancer therapeutics that target Cyt*c* for the first time.

## Figures and Tables

**Table 1:** Crystallographic data summarizing and comparing three structures of cytochrome *c*: Native, K53Q, and human Y46F.

Structure	Cyt*c* Mouse	Cyt*c* (K53Q) Mouse	Cyt*c* (Y46F) Human
**PDB code**	**5C0Z**	**7LJX**	**3ZOO**
**Data code**	141,118 × 21a	161,109 × 06a	
**CRYSTALLIZATION**			
**Iron**	Oxidized	Oxidized	Oxidized
**Protein**	15 mg/mL Cyt*c* (WT) + 5 mM K_3_Fe(CN)_6_ in water	17mg/mL Cyt*c* (K53Q) + 5 mM K_3_Fe(CN)_6_ in water	12.5 mg/mL oxidized Cyt*c* in 22.5% (*w*/*v*) PEG-1000, 50 mM
			KH_2_PO_4_, pH 7.0
**Well**	25% PEG 4K, 8% isopropanol, 0.1 M Na Acetate, pH 6.5	30% PEG 2K MME, 0.1M Na MES, 0.1 M Na Acetate, pH 6.5	26–31% (*w*/*v*) PEG 1000, 40 mM, KH_2_PO_4_. pH 7.0
**Drop**	1:1 Protein:Well	1:1 Protein:Well	n/a
**Cryoprotectant**	30% PEG 4K, 8% isopropanol, 0.1 M Na Acetate, 20% Ethylene glycol, 10 min soak with 5 mM K_3_Fe(CN)_6_, final pH 6.5	35% PEG 2K MME, 0.1M Na MES, 0.1 M Na Acetate, pH 6.5, 20% Ethylene glycol	26–31% (*w*/*v*) PEG 1000, 40 mM, KH_2_PO_4_, 15% Glycerol, pH 7.0
**CRYSTAL DATA**			
**Space group:**	P1	P2(1)2(1)2	P1
**Unit cell: a**	34.401	52.837	36.367
**b**	52.471	96.767	53.952
**c**	61.647	38.372	58.95
**Alpha**	110.04	90	76.55
**Beta**	92.77	90	88.73
**Gamma**	92.02	90	71.86
**Chains per A.U.**	4	2	4
**Matthews Coeff**	2.24	2.1	2.3
**Solvent %**	45.12	41.4	46.7
**X-RAY DATA**			
**Resolution-high (Å)**	1.12	1.31	1.35
**Resolution-low (Å)**	49.22	48.38	30.59
**Beamline**	APS 21-ID-F	APS 21-ID-D	DIAMOND 103 (UK)
**Wavelength**	0.97872	1.07822	0.9762
**Reflections**	127840	48257	81707
**Completeness**	87.4 (44.7)	98.9 (86.8)	94.7 (80.7)
**Average I/sigma**	14.4 (2.0)	12.2 (2.1)	8.70 (n/a)
**Redundancy**	3.9 (3.7)	11.1 (6.6)	n/a
**Resolution-high (Å)**	1.12	1.31	1.35
**Resolution-low (Å)**	49.22	48.38	30.59
**Rmerge**	0.050 (0.548)	0.120 (0.821)	0.07
**REFINEMENT**			
**Rfactor**	0.132 (0.239)	0.138 (0.291)	0.138
**Rfree**	0.159 (0.240)	0.170 (0.316)	0.179
**Avg B-factor (Å2)**	15.97	21.32	17.703
**Protein atoms per A.U.**	3228	1614	3981
**Water molecules**	537	194	417
**Bond RMSD**	0.017	0.016	0.017
**Angle RMSD**	1.903	2.276	1.99
**FC6 OCCUPANCY**			
**202a/A**	0.28	0.5	
**202b/A**	0.37		
**202/B**	0.93		
**203/B**	–	–	
**203a/B**	0.3		
**203b/B**	0.32		
**202/C**	0.9		
**HEME LINKS:**			
**Average (Å)**			
**C14 SG—HEM CAB**	1.90 (0.04)	1.77 (0.01)	1.95 (0.02)
**C17 SG—HEM CAC**	1.98 (0.04)	1.86 (0.00)	2.13 (0.07)
**H18 NE2—FE2 (Å)**	2.02 (0.01)	1.99 (0.01)	2.02 (0.03)
**M80 SD—FE2 (Å)**	2.30 (0.01)	2.31 (0.01)	2.28 (0.02)

## Data Availability

Structural data have been posted under the PDB code 7LJX (http://www.rcsb.org (accessed on 5 March 2021)).

## Abbreviations

COX	cytochrome *c* oxidase
Cyt*c*	cytochrome *c*
ETC	electron transport chain
OxPhos	oxidative phosphorylation
ROS	reactive oxygen species
